# Acute and medium term effects of a 10-week running intervention on mood state in apprentices

**DOI:** 10.3389/fpsyg.2013.00411

**Published:** 2013-07-09

**Authors:** Katrin Walter, Birte von Haaren, Simone Löffler, Sascha Härtel, Carl-Philipp Jansen, Christian Werner, Jürgen Stumpp, Klaus Bös, Stefan Hey

**Affiliations:** ^1^House of Competence - hiper.campus, Karlsruhe Institute of TechnologyKarlsruhe, Germany; ^2^Department of Sport and Sports Science, Karlsruhe Institute of TechnologyKarlsruhe, Germany

**Keywords:** mood, activity intensity, ambulatory assessment, aerobic exercise, randomized controlled trial, inactive people

## Abstract

Exercise and physical activity have proven benefits for physical and psychological well-being. However, it is not clear if healthy young adults can enhance mood in everyday life through regular exercise. Earlier studies mainly showed positive effects of acute exercise and exercise programs on psychological well-being in children, older people and in clinical populations. Few studies controlled participants' physical activity in daily life, performed besides the exercise program, which can impact results. In addition the transition from mood enhancement induced by acute exercise to medium or long-term effects due to regular exercise is not yet determined. The purpose of this pilot study was to examine the acute effects of an aerobic running training on mood and trends in medium term changes of mood in everyday life of young adults. We conducted a 10-week aerobic endurance training with frequent mood assessments and continuous activity monitoring. 23 apprentices, separated into experimental and control group, were monitored over 12 weeks. To control the effectiveness of the aerobic exercise program, participants completed a progressive treadmill test pre and post the intervention period. The three basic mood dimensions energetic arousal, valence and calmness were assessed via electronic diaries. Participants had to rate their mood state frequently on 3 days a week at five times of measurement within 12 weeks. Participants' physical activity was assessed with accelerometers. All mood dimensions increased immediately after acute endurance exercise but results were not significant. The highest acute mood change could be observed in valence (*p* = 0.07; η^2^ = 0.27). However, no medium term effects in mood states could be observed after a few weeks of endurance training. Future studies should focus on the interaction between acute and medium term effects of exercise training on mood. The decreasing compliance over the course of the study requires the development of strategies to maintain compliance over longer periods.

## Introduction

Several studies have shown that regular exercise and physical activity increased emotional well-being. Ross and Hayes (1988) as well as Stephens (1988) demonstrated that physical activity improved mood, and symptoms of depression and anxiety could be reduced. Research conducted to examine the effects of exercise on mood addressed either acute effects of exercise on mood or effects of aerobic exercise programs on mood. Acute exercise of low and moderate intensity showed the highest effects on mood while there was more variability between individuals' mood states during and after exercise of high intensity (Ekkekakis et al., 2011; Reed and Ones, 2006).

Several randomized controlled trials showed that regular aerobic exercise of low intensity as well as moderate and high intensity improved positive affect (Reed and Buck, 2009). In both acute and regular exercise, people with lower baseline mood values reported higher mood changes due to exercise (Reed and Ones, 2006; Reed and Buck, 2009).

Despite several studies showing benefits for mood induced by regular exercise, reasons remain undetermined yet. Reed and Buck (2009) reviewed different explanations of several authors. Mood effects due to regular exercise might be induced by the accumulation of acute effects induced by exercise. Repeated exercise may result in stress adaptation and thus improve affect. Alternatively, acute exercise improves positive affect and observed changes in combination with regular exercise reflect the repeated acute affective improvement. The maintenance explanation is supported by the aspect that according to Ekkekakis et al. (2000) affective changes induced by exercise are independent of fitness changes. Acute effects are observed regardless of training response. One would expect higher effects of regular exercise on mood compared to acute effects, but reviews only showed slight differences in effect size (Reed and Buck, 2009). In addition, Steinberg et al. (1998) found similar instead of increasing acute mood effects every week over the course of his study.

Most former studies examining the effect of regular exercise on mood focused on clinical or older populations. Samples including young adults mainly considered student samples. In addition, these studies did not control daily life physical activity besides the exercise intervention which might impact the results. In addition to progressive exercise tests to check the effectiveness of the exercise intervention, objective activity monitoring allows for important continuous insights into physical activity behavior of both control and experimental group pre, during and post the intervention.

Former studies mainly conducted exercise programs assessing affect pre and post the exercise program using single occasion retrospective self-report measures. In general assessing emotional states by means of retrospective reports holds the risk of systematic recall biases. During self-reporting, subjects typically use a variety of heuristics, namely not only retrieving their emotional state, but also aggregating and summarizing experiences (Cohen and Java, 1995; Hufford et al., 2001; Fahrenberg et al., 2007; Shiffman et al., 2007).

The positive effects of regular exercise on mood are probably expected by most of the society. The social desirability of reporting higher mood states if asked once after an exercise intervention may have led to relatively constant results showing mood improvement due to regular exercise in the past.

In general, ecological momentary assessment allows for real-time assessment of mood to increase accuracy and reduce retrospective biases. In addition, the assessment in real-life situations enhances generalizability and the repeated assessments allow for the investigation of dynamic processes. In this study, EMA was used to get real time data immediately before and after exercise and to examine medium term variations over the whole study period.

With the growing interest of the effects of physical activity on mood in daily life, some recent studies have addressed this question. Kanning and Schlicht (2010) observed increased mood after self-reported physical activity episodes. Two studies using ambulatory assessment showed that an increase in physical activity induced an increase in positive affect in everyday life (Schwerdtfeger et al., 2008; Kanning et al., 2012).

To our best knowledge no study analyzed the interaction between acute and medium term effects of acute and regular aerobic exercise on mood in everyday life of apprentices. Only one study investigated mood more frequently (weekly basis) during an exercise intervention period. In addition, no study examining effects of regular aerobic exercise on mood in everyday life controlled daily life physical activity besides the exercise intervention. With the present pilot study we wanted to check if enhanced mood due to acute exercise transfers to increased mood trends in daily life induced by regular exercise. In addition, we were interested if more frequent mood measures in daily life during an exercise program confirm the results of earlier studies.

To capture more situations of acute changes in mood due to exercise and to better understand the interaction between acute and medium term effects of exercise by measuring mood more frequently during the exercise intervention, we addressed this question using ambulatory assessment. To control daily life physical activity besides the exercise intervention, we objectively monitored physical activity continuously via accelerometry. In addition, we expected to get insights into the activity behavior of both groups during different phases of the study. To secure the effectiveness of the aerobic endurance program, a sample of apprentices was chosen which was not engaged in aerobic endurance training before. As established in the literature, the highest improvements in fitness can be achieved in participants with low baseline fitness level (Zintl, 1997).

We hypothesize that acute aerobic endurance exercise induces increases in mood in young adults. We further estimate that regular exercise leads to medium term effects reflected by increased mood in everyday life.

## Method

### Participants and procedure

Participants were recruited from the age group of apprentices who started at Karlsruhe Institute of Technology (KIT) in 2010. Due to an information letter from the KIT vice president and an information event, 25 apprentices gave written informed consent to participate in the study. Participants were divided into control and experimental group by lot based on stratification of gender to balance the distribution in both groups.

23 participants (12 f, 11 m) were included in the analyses, as two participants of the experimental group dropped out due to injury or motivational reasons. Due to the stratification of gender, the percentage of male and female in the experimental group (EG: ♀ = 6; ♂ = 6) and the control group (CG: ♀ = 6; ♂ = 5) had a similar distribution. The average age of all participants was 19.43 (±1.85) years. The participants of the *EG* were on average 6 months younger (19.17 ± 1.47 years) than those of the *CG* (19.73 ± 2.24 years).

**Figure 1:**
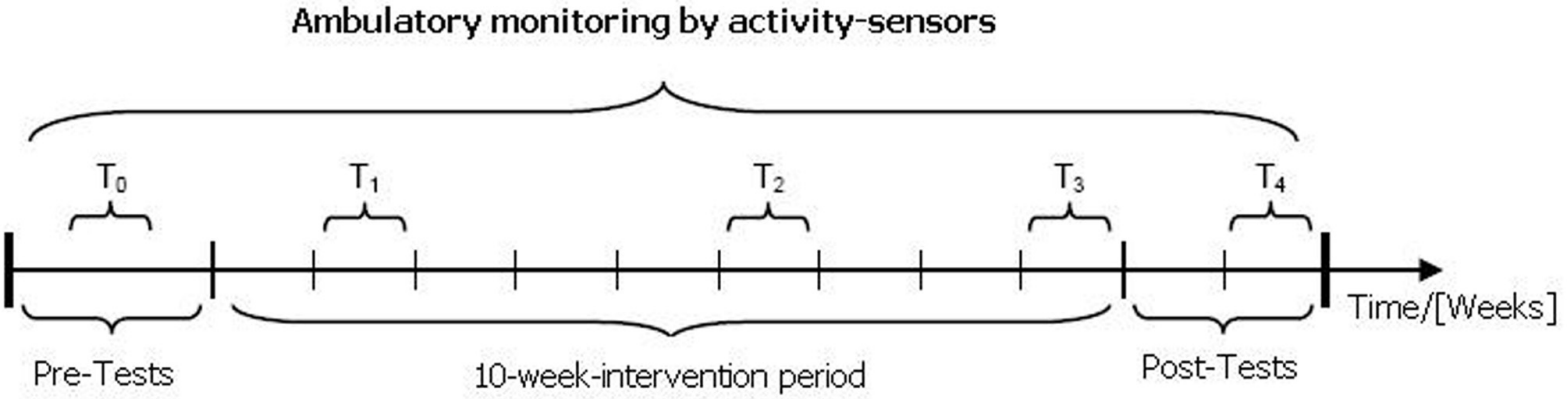
**Study design (Test of aerobic endurance capacity at T0 and T4, assessment of activity and mood at T0–T4)**.

All data of this pilot study was assessed during a period of 8 months between September and April. All pre- and post-tests (T0 and T4: see Figure 1) were carried out in the outpatient medical center and at the Institute of Sport and Sports Science of the KIT. Since there was not sufficient time to carry out the pre-tests with the entire sample before the start of the intervention, few participants of the control group were tested after the start of the intervention. Baseline mood assessment (T0) was conducted before the start of the intervention.

An aerobic endurance intervention was conducted over a period of 10 weeks during winter months. Participants of the *EG* had to participate in an instructed outdoor running training twice a week. The *CG* participants were instructed not to alter their physical activity and exercise patterns during the control period. During the intervention/control period participants' mood states were recorded using electronic diaries on three consecutive days during the second (T1), sixth (T2), and tenth (T3) week (see Figure 1). After the 10-week intervention period the post-tests of aerobic endurance capacity and a final monitoring of mood states were conducted (T4: see Figure 1) as a retention measurement to capture possible long-term effects in the twelfth week. Additionally, mood ratings pre and post the endurance training were conducted in the experimental group. Physical activity was monitored continuously during the whole study. A detailed description of measurement procedures is given in the following section.

## Measurement procedures

The pre- and post-tests to assess endurance capacity took place at T0 and T4. The data acquisition of subjective variables (via electronic diaries) and objective variables (via accelerometry) was conducted at T0 and T4 as well as during the 10-week intervention/control period (T1–T3).

### Test of endurance capacity

To assess the aerobic endurance capacity, a progressive treadmill test to exhaustion with gas analyses was conducted. A 6-2-3 protocol (6 km/h initial load − 2 km/h increment per stage − 3 min duration of stage) was used (Dickhuth et al., 2007).

Since the participants were not experienced in running on a treadmill and had not performed a treadmill test before, they were instructed to warm up by running slowly for a short period (1–2 min) in order to get used to the treadmill and to practice the straddling position on the outer surfaces of the treadmill (to facilitate the acquisition of blood samples). After the warm up period, the test started at the initial speed of 6 km/h. At the end of each stage, the heart rate was recorded using POLAR® heart rate monitors, model “RS 800.” Within a standardized break of 20 s between stages, an arterial blood sample was taken from the participants' earlobe into a 20 μl end-to-end capillary for the analysis of blood lactate.

For the design of the endurance training, the main outcome variables were running speed at 4 mmol/l lactate, at the lactate threshold (LT), at the individual anaerobic threshold (IAT) and the maximum speed (see Heck et al., 1985). Based on the LTs, the individual heart rate zone was determined using the software Ergonizer® (Sports medicine Freiburg, K. Röcker).

### Assessment of mood states

To assess mood states frequently in everyday life, the short-scale developed by Wilhelm and Schoebi (2007) to measure the three basic mood dimensions valence, calmness and energetic arousal, was used. The scale incorporates only two items per mood dimension. The participants had to respond to the statement “At the moment I feel … ” by providing information relating to six bipolar items, which were each graded on a seven-step scale (0–6) with the contrary adjectives as endpoints. Energetic arousal was assessed by the items tired-awake and full of energy-without energy; calmness by agitated-calm and relaxed-tense and valence by content-discontent and unwell-well.

Each dimension was, therefore, ascribed both a negatively and positively polled item. In order to obtain rectified scale values for each dimension, the items relating to the negative mood pole (“content-discontent,” “full of energy-without energy,” and “relaxed-tense”) were recoded. Thus, low values indicated low calmness, valence and energetic arousal and higher values indicated a higher level of the three mood dimensions. The mean value per dimension was used for statistical analysis (Wilhelm and Schoebi, 2007).

To be able to assess changes of mood over time, the recording tool has to have sufficient sensitivity to change. The sensitivity to change relates to the “characteristic of a psychological test value [.], to show real changes, i.e., mapping not only measurement errors but functionally important adjustment processes within the variability of a time series” (Fahrenberg et al., 2002). Wilhelm and Schoebi (2007) demonstrated that the three dimensions of the short-scale have a high sensitivity to change and therefore are deemed a suitable measuring tool for recording changes in mood over time. Intra-individual reliability rates (r.70 for valence and calmness, *r* = 0.77 for energetic arousal) provide satisfactory internal consistency.

The assessment of mood states via electronic diaries was carried out at each time of measurement (T0– T4) on 3 days per week: Tuesday, Wednesday, and Thursday. Since mood state on Mondays and Fridays might be influenced by past or forthcoming events at the weekend. Furthermore, the KIT apprentices sometimes did not follow their normal daily work routine on Fridays due to instruction at the vocational training school. Thus, the collection of data was avoided on these days.

My Experience movisens Edition (movisens GmbH, Karlsruhe) software was used to install the mood-scale (Wilhelm and Schoebi, 2007) on a PDA (personal digital assistant; HTC Touch Diamond 2) and to program the time intervals between the daily queries. Over the course of the day, the participants were reminded via a vibrating and/or an acoustic signal to answer the queries via touch screen at the specified times (after getting up in the morning, 10 am, 1 pm, 4 pm, 8 pm). In addition, the participants of the *EG* had to answer the questions at three given times after the training session, immediately after the end of the training, 20 and 40 min after the training session. To avoid anticipation of the signal and thus the possibility of associated changes in behavior, a random component of ±15 min was chosen. If the study participant did not respond immediately, the signal was repeated at 5, 10, and up to 15 min after the original request. If the last request was not answered within 5 min, the request was labeled as a missing value.

### Activity monitoring

Participants' physical activity was recorded with the Move II accelerometer (movisens GmbH, Karlsruhe) attached to the hip. The Move II is a light weight sensor (17 g; 50 × 36 × 17 mm) recording raw acceleration data by means of 3-axis (64 Hz, ±8 g, 12 bit, 4 mg) with battery life of up to 7 days. The sensor had to be replaced each week in order to obtain data for the entire study period. The sensors were individually configured via an USB port for each participant (according to age, sex, height, and weight) with the appropriate software (SensorManager, UnisensViewer and DataAnalyzer, movisens GmbH, Karlsruhe). The recorded acceleration raw data was saved on a micro SD card. The raw data after a complete measurement was transmitted to a computer via an USB 2.0 interface. Mathematical and statistical procedures applied to the raw data can be used to differentiate between several activities: rest (standing, lying, or sitting), cycling/ergometer, climbing stairs, walking (jogging, slow, or normal walking), and “unknown activities” (for details see Härtel et al., 2011). For this study the raw data captured by the accelerometer was used to calculate the activity intensity in mg.

### Aerobic endurance intervention

The *EG* had to complete an instructed aerobic running training session twice a week over a period of 10 weeks. To achieve good compliance, three instructed running sessions per week at suitable times during apprentices' daily routine were offered. The running sessions took place in a natural outdoor environment. Most sessions were held in the afternoon and few during lunch breaks.

Based on the LT and the IAT, individual training heart rate zones (THRs) were determined for the *EG* participants (Dickhuth et al., 2007). The range within heart rate zones was established as follows:

The *EG* participants were fitted with the POLAR® Modell “RS800” heart rate monitors which they wore throughout the entire intervention period and which were used to monitor individual THRs during training sessions. To ensure that participants practiced within their individual THR, the heart rate monitors were programmed to emit an acoustic signal to increase or reduce running speed if heart rate was below or above the individual THR. In the exceptional case that a participant had to complete a running session on his/her own, completion of this session was monitored by the heart rate monitors. To develop the basic aerobic endurance, a continuous endurance method suitable for beginners was applied to the experimental group. The initial duration of 30 min was continuously increased to 60 min over the 10 weeks. Besides the increase of duration, intensity was progressively increased by adding short intervals of 2 min above the individual THR after week 6. As part of a variable endurance method, the aerobic-anaerobic functional range was improved (Kubukeli et al., 2002; Tanisho and Hirakawa, 2009).

## Data analysis

Only *EG* participants who had completed at least 50% of the prescribed 20 training sessions were included in the analysis. One female participant did not achieve this requirement and was therefore excluded from the statistical analysis. We assumed that participants who completed fewer training sessions during the 10-week intervention period couldn't achieve a training effect.

The statistical analysis of all group differences was conducted with a maximum of *n* = 22 participants (*EG* = 11; *CG* = 11). Two *CG* participants did not engage in the post-test of endurance capacity at T4. Thus, the sample size for the analysis of the pre and post-endurance capacity was reduced to *n* = 20 (*EG* = 11; *CG* = 9).

### Short-term effects of acute exercise on mood

To analyze short term effects of the endurance training on current mood, the queries which were answered immediately prior to and immediately after the training session were considered. For the mood values prior to the training only data collected within the last hour before the training was considered. It was assumed that earlier queries did not reflect the current mood state of participants before the training. Due to a high number of missing values prior to and immediately after training, only 37 out of possible 72 data entries (two training sessions per time of measurement T1–T3; *n* = 12 for EG) were available for analysis. According to earlier studies a mean value of each *EG* participant's completed queries before and immediately after the training was calculated (Alfermann and Stoll, 1996).

### Medium-term effects of regular exercise on mood

To analyze medium term effects of the exercise intervention on mood (recorded via electronic diaries) data of participants who completed at least six queries at each time of measurement (T0–T4) on at least 2 days and more than 1/3 of all queries was included. Due to the additional three queries after the training sessions, each participant of the *EG* had a maximum number of 21 data entries per week if all queries were answered.

The compliance to mood assessments of experimental and control group was calculated as sums for each time of measurement to analyze the change over time. Table 3 shows the answered queries for *EG* and *CG* at every time of measurement (sum), the maximum number of queries (max poss), and the actual compliance as the rate of answered queries (percentage), overall and separated for *EG* and *CG*, respectively at T0–T4 (Stone et al., 2003).

### Mood-dimensions

To analyze the mood dimensions mean daily (DV) and weekly values (WV) were calculated for each mood dimension (calmness, valence, and energetic arousal). The daily value was calculated as the mean of the daily queries for each participant and dimension. For each time of measurement (T0–T4), a weekly value was calculated as the mean value of the three daily values.

To analyze the short-term effects of training sessions on current mood, a mean value for all available data entries prior to the training sessions (MVpre) was calculated for every participant and dimension. Second a mean value for all available data entries immediately after the training sessions (MVpost) was calculated for each participant and dimension. The number of data entries included in the calculation of the mean values differed considerably among participants. For some participants, up to seven data entries were available while for others only two were available.

### Activity intensity

Participants' physical activity was recorded via accelerometry during the entire study period. The raw acceleration data captured by the accelerometer (acceleration in g) was used to calculate the activity intensity in mg. Here, activity intensity is the average amount of acceleration per minute. If activity intensity fell below a threshold of 20 mg for at least 1 h, it was defined as “non-wearing time.” The non-wearing time was compared to the 24 h during which the sensor could be worn as maximum wearing time per day (off ratio). Values below the minimum wearing time of 8 h per day or an off ratio >67% were not considered for analysis.

The time spent in different activity intensity levels (see Table 2) was calculated for the pre- and post-intervention period as well as during the intervention period. Based on the individual daily values, overall and group specific values were calculated. For the pre-intervention period (pre), the mean value of 7 days of acceleration data was calculated. For the intervention period (int), all daily values during the 10 weeks intervention period were averaged. Post-intervention values (post) were calculated based on 7 days of activity assessment after the intervention period. Due to technical errors and missing data during the intervention period, the number of days included in the analysis varied from 7 to 62 across participants.

## Results

### Physical activity behavior

It was assumed that each participant wore (more or less) the accelerometer for at least 8 h per day, excluding sleeping time and the time in which the sensor was taken off for technical reasons (showering, swimming, etc.).

In addition, participants were instructed to take off the sensor during the night. Nevertheless a very few participants apparently continued to wear the sensor during sleep. If a participant had an off-ratio <10% it was assumed that the sensor was worn during sleeping time. Thus, days with an off-ratio <10% were excluded from analysis to get comparable results across participants.

A minimum wearing time of 7 days (10080 min) was required for each period of activity assessment [pre, during (int), and post-intervention period]. During the intervention period, wearing time varied extremely across participants. Some participants achieved the required minimum of at least 7 days; others wore it up to 62 days in total. The calculation of wearing time only included “active wearing time” during daytime.

While the wearing time was almost equal before the intervention period in both groups (*CG* = 46%; *EG* = 44%; 10080 min reflecting 7 days = 100%), the experimental group wore the sensor significantly more during intervention period (*EG* = 266%; *CG* = 148%). While the *EG* was in contact with the principal investigator twice a week due to training sessions, making appointments with participants of the *CG* to exchange the accelerometers was not feasible.

Participants' wearing time decreased in both groups from pre to post-intervention (−15% wearing time). While participants wore the accelerometer about 73 h during the pre-intervention period, it was worn not more than approximately 55 h in *EG* and 40 h in *CG* during the post-intervention period.

The physical activity level (mean activity intensity) analyzed over the entire intervention period (pre, int, post) differed between the control and experimental group. The analysis of variance for mean activity intensity showed a significant interaction within “group × time” [*F*_(2, 36)_ = 3.40; *p* = 0.04; η^2^ = 0.16].

Mean activity intensity (mean mg value, Table 1) prior to the intervention (pre) was similar in both groups (difference not significant). While mean intensity level tended to decrease from prior (pre) to during the intervention (int) in the *CG*, it increased significantly in the *EG* (*t* = −3.35; df = 9; *p* = 0.008). In the post-intervention period, the *CG* showed a similar activity intensity level as before the intervention period (pre). The activity intensity of the *EG* tended to decrease after the intervention period (post) but was still on a higher level than before the intervention period (n.s).

**Table 1:** **Mean activity intensity (mg) for *EG* and *CG* during the three periods (pre, int, and post) of activity monitoring**.

	**Mean activity intensity in mg (±*SD*)**		***n***	
	***EG***	***CG***	***EG***	***CG***
pre	83.9 (±33.4)	82.5 (±17.6)	10	10
int	98.0 (±15.3)	78.0 (±16.1)	10	10
post	87.5 (±23.7)	81.0 (±27.1)	10	10

Both groups spent most of their time in the lowest intensity-level (0–99 mg) during all three periods of activity measurement. No significant changes could be observed for the time spent in different activity intensity levels from pre to int and post. The *EG* increased their time spent in the highest intensity level (1000–9999 mg) from 0 to 1% during the intervention period illustrating the intensity of a running training (Kanning et al., 2012), but showed a decrease post the intervention period (0.2%) (Table 2).

**Table 2:** **Percentage of minutes spent in different activity intensity levels (mg) pre, during, and post the intervention period for *CG* and *EG***.

	**Minutes in total (min)**		**Minutes spent in activity intensity level (AL in mg) in percent (%)**												
	***EG***	***CG***	**AL 0–99 mg**		**AL 100–199 mg**		**AL 200–299 mg**		**AL 300–399 mg**		**AL 400–499 mg**	
			***EG***	***CG***	***EG***	***CG***	***EG***	***CG***	***EG***	***CG***	***EG***	***CG***
pre	844.7	831.0	75.2%	76.8%	13.1%	13.9%	5.5%	4.8%	2.7%	2.0%	1.8%	1.1%
int	808.2	816.3	73.3%	75.8%	13.7%	14.4%	5.7%	4.8%	2.6%	2.1%	1.6%	1.3%
post	828.1	814.2	73.7%	75.7%	14.1%	13.8%	5.8%	5.0%	2.9%	2.5%	1.7%	1.6%
**Minutes spent in activity intensity level (AL in mg) in percent (%)**												
	**AL 500–599 mg**		**AL 600–699 mg**		**AL 700–799 mg**		**AL 800–899 mg**		**AL 900–999 mg**		**AL 1000–9999 mg**	
	***EG***	***CG***	***EG***	***CG***	***EG***	***CG***	***EG***	***CG***	***EG***	***CG***	***EG***	***CG***
pre	1.1%	0.7%	0.4%	0.5%	0.2%	0.1%	0.1%	0.0%	0.1%	0.1%	0.0%	0.1%
int	1.0%	0.8%	0.5%	0.5%	0.2%	0.2%	0.1%	0.1%	0.1%	0.0%	1.0%	0.3%
post	0.9%	0.9%	0.4%	0.5%	0.2%	0.3%	0.2%	0.2%	0.0%	0.2%	0.2%	0.6%

### Change in endurance capacity

To test if endurance training was effective and thus participants' endurance capacity improved from T0 to T4, the following parameters were taken into account: running speed at 4 mmol/l (v4 mmol/l), at the vLT, at the vIAT and at the time the test was terminated (vmax). *EG* increased their running speed from T0 to T4 for all parameters [v4 mmol/l (+0.58 km/h), vLT (+0.10 km/h), vIAT (+0.38 km/h), vmax (+0.85 km/h)]. In contrast the *CG* showed a slightly reduced running speed for these parameters (range of reduction: 0.11 km/h to 0.36 km/h) at T4.

The repeated measures analysis of variance revealed a significant interaction between time and group for v4 mmol/l [*F*_(1, 18)_ = 4.74; *p* = 0.04], vIAT [*F*_(1, 18)_ = 9.02; *p* = 0.01] and vmax [*F*_(1, 18)_ = 7.59; *p* = 0.01]. The effect size for these parameters ranged from 0.21 to 0.33.

## Compliance to mood assessments

Prior to the intervention period (T0) more than 2/3 of all possible queries were answered by both *EG* (72.22%) and *CG* (70.90%). At the end of the study (T4) *CG* answered less than half (47.27%) and *EG* answered even less than 1/3 (30%) of the maximum possible queries (see Table 3).

**Table 3:** **Compliance to mood assessments at the different times of measurement (T0–T4)**.

**Queries**	**T0**			**T1**			**T2**			**T3**			**T4**		
	***EG***		***CG***	***EG***		***CG***	***EG***		***CG***	***EG***		***CG***	***EG***		***CG***
Sum	130		117	133		87	109		95	99		77	54		78
Max poss	180		165	252		165	252		165	252		165	180		165
Percentage	72%		71%	53%		53%	43%		58%	39%		47%	30%		47%
Sum (*CG* + *EG*)		247			220			204			176			132	
Max poss (*CG* + *EG*)		345			417			417			417			345	
Percentage (*CG* + *EG*)		72%			53%			49%			42%			38%	

A repeated measures analysis of variance revealed significant differences for the factor “time of measurement” [*F*_(4, 0)_ = 3.19; *p* = 0.04; η^2^ = 0.42]. *Post-hoc* analyses showed that compliance was significantly reduced from T0 to T3 (*t* = 2.83; *df* = 22; *p* = 0.01).

### Change in mood—acute effects

Due to insufficient compliance for the mood ratings pre, directly after and 20, 40 min post-training, acute effects after training could only be analyzed pre and post-training. In addition, acute effects due to exercise training could not be calculated for training sessions separately for each time of measurement. Thus, acute mood effects pre and immediately after training (post) were calculated both for T1 and averaged over T1–T3 (intervention period).

### Acute effects at T1

Immediately after the training, participants rated 10.38% higher values of energetic arousal than before the training. Valence (−8.68%) and calmness (−9.97%) both decreased from pre to post-training. The analysis of variance revealed no significant differences between pre and post-training in energetic arousal [*F*_(1, 9)_ = 1.93; *p* = 0.199; η^2^ = 0.18], valence [*F*_(1, 9)_ = 1.74; *p* = 0.22; η^2^ = 0.16], and calmness [*F*_(1, 9)_ = 3.27; *p* = 0.10; η^2^ = 0.27].

### Acute effects across the intervention period (T1–T3)

Immediately after the training participants, on average, rated higher scale values for all three dimensions compared to prior to the training (*E* = + 10.6%; *V* = 17.8%; *C* = 6.2%). The most distinct change could be observed in valence. While mean valence was 3.48 (±0.57) prior to the training, the participants achieved an average scale value of 4.10 (±1.10) immediately after the training. The smallest changes (+6.2%) from pre (*C*: 3.73 ± 1.24) compared to post (*C*: 3.96 ± 0.58) training session were observed in calmness.

The analysis of variance revealed a marginally significant difference between the scale values of valence pre and post-training sessions [*F*_(1, 11)_ = 3.99; *p* = 0.07; η^2^ = 0.27]. No significant differences could be identified between the two times of measurement for energetic arousal—[*F*_(1, 11)_ = 0.80; *p* = 0.39; η^2^ = 0.07] and calmness [*F*_(1, 11)_ = 1.19; *p* = 0.30; η^2^ = 0.09].

### Change in mood—medium-term effects

Due to the significantly decreasing compliance at T3 and T4 resulting in a smaller sample size, inferential statistics could not be conducted to analyze medium term effects of the 10-week endurance training on mood in daily life. *EG* participants report a continual reduction of all dimensions across the five times of measurement except for energetic arousal, which rises between T2 and T3. In contrast enhanced mood values of energetic arousal, valence, and calmness were observed for the *CG* at the end of the study. Due to insufficient data available at T4 (see Table 4), the mean values and standard deviations of the three mood dimensions C, V, E are shown descriptively for the five times of measurement (see Table 4).

**Table 4:** **Mean scale values of the mood dimensions *E*, *V*, *C* at T0–T4, respectively for *EG* and *CG***.

	**T0**		**T1**		**T2**		**T3**		**T4**	
	***EG***	***CG***	***EG***	***CG***	***EG***	***CG***	***EG***	***CG***	***EG***	***CG***
E_MV	3.82	3.41	3.44	3.96	3.22	3.82	3.55	3.32	3.35	3.91
E_SD	0.86	0.81	1.21	0.89	0.77	0.87	0.86	0.77	0.87	0.74
V_MV	4.14	4.24	4.02	4.55	3.87	4.52	3.84	4.38	3.64	4.56
V_SD	0.74	0.71	0.83	0.82	0.93	0.7	0.78	0.5	0.75	0.65
C_MV	4.1	4.37	3.87	4.47	3.9	4.48	3.8	4.36	3.74	4.52
C_SD	0.63	0.71	0.37	0.75	0.76	0.58	0.73	0.44	0.72	0.65
*n*	9	9	10	8	9	8	6	6	4	5

To be able to calculate inferential statistics, the mood ratings of T2 and T3 were summed to obtain a larger sample size. We selected the time of measurement with the highest number of data entries, if a minimum of six queries was answered. The resulting sample sizes are presented in Table 5.

**Table 5:** **Differences in mood dimensions *E*, *V*, *C* (mean scale values) between T0 and T2/3 for both groups**.

	**T0**		**T2/3**		**Diff**		**Diff%**	
	***EG***	***CG***	***EG***	***CG***	***EG***	***CG***	***EG***	***CG***
E_MV	3.25	3.08	3.17	3.1	−0.09	0.02	−2.77	0.65
E_SD	0.279	0.296	0.307	0.260	0.47	0.34		
V_MV	3.13	3.22	3.05	3.04	−0.08	−0.19	−2.56	−5.89
V_SD	0.430	0.223	0.116	0.350	0.43	0.44		
C_MV	3.25	3.15	3.16	3.22	−0.09	0.07	−2.77	2.23
C_SD	0.323	0.304	0.346	0.171	0.26	0.25		
*n*	9	8	9	8	9	8	9	8

The *T*-test revealed no significant differences for the mood dimensions at T0 and T2/3. The comparison of mood changes from baseline to T2/3 showed decreasing values of the *EG* for all three dimensions. In the EG, C scale values decreased by 2.8% while they increased in the *CG* [*t*_(15)_ = 0.45; *p* = 0.23]; (Cohen's d = −0.62). A similar result was found for the E dimension [*t*_(15)_ = 0.81; *p* = 0.59]; (Cohen's *d* = −0.27). Valence decreased by 2.56% in the *EG* and by 5.89% in the *CG* [*t*_(15)_ = 0.61; *p* = 0.63]; (Cohen's *d* = 0.25).

## Participants with good compliance

**Figure 2:**
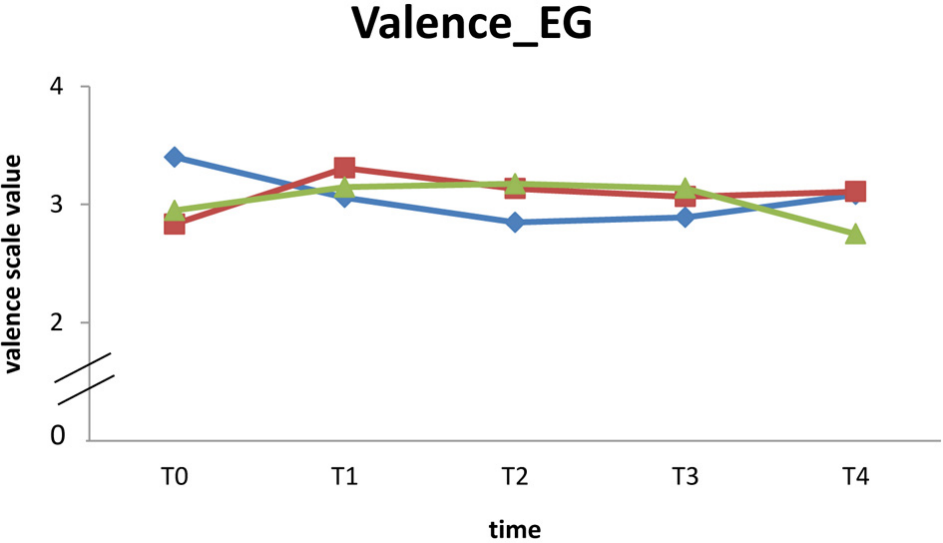
**Individual trend of valence from T0 to T4 of participants (*EG*) with good compliance**.

**Figure 3:**
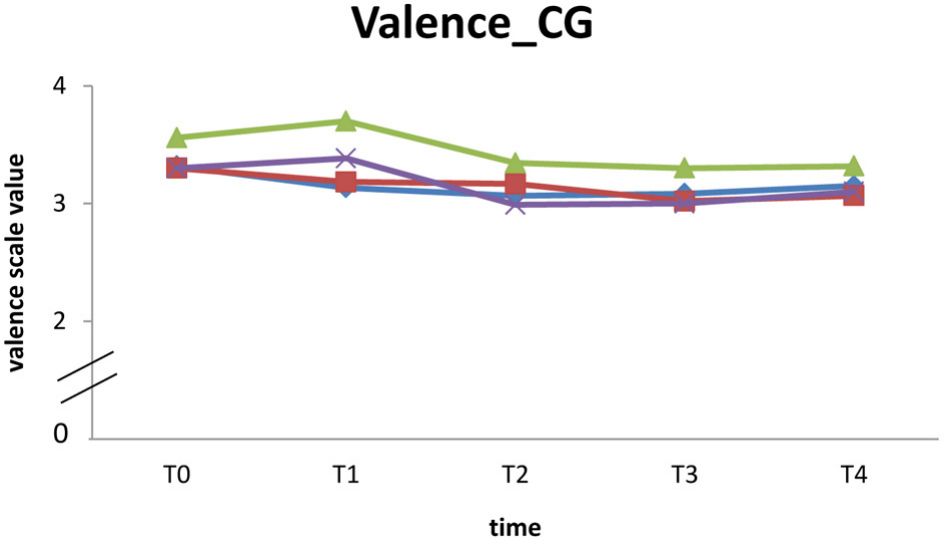
**Individual trend of valence from T0 to T4 of participants (*CG*) with good compliance**.

No inferential statistics was calculated for medium term effects due to significantly decreasing compliance at T3. Seven participants showed good compliance answering at least eight queries at every time of measurement. For these participants (EG: *n* = 3; *CG*: *n* = 4) individual trends are displayed for the valence dimension (highest increase in examination of acute effects compared to other mood dimensions) in Figure 2 (*EG*) and Figure 3 (*CG*).

## Discussion

In the present ambulatory assessment study we examined acute effects and medium term effects of endurance exercise on mood states in everyday life of apprentices during a 10-week endurance intervention.

### Acute effects of exercise

At the beginning of the in intervention period (T1) only energetic arousal tended to increase immediately after training, whereas the valence and calmness values tended to decrease.

Due to the low compliance of apprentices to rate mood states five times a day, our intention to examine acute effects of training on a weekly basis was not feasible. Intra- individual differences as supposed to exist in previous studies (Ekkekakis et al., 2011) were not considered due to the high number of missing data. Despite the fact that most participants completed the acute mood assessments pre and post the training we could not show significant effects with the small sample size. With averaging all assessments of acute effects of training on mood dimensions we showed marginally significant effects for valence. In contrast to earlier studies mood state pre and post-training was assessed more frequently to test if effect sizes of acute effects increased over the course of the intervention. By averaging multiple acute assessments more situations are captured but inter and intra-individual variability may cover the tracks of real effects.

However, for the mean values across the whole intervention period (T1–T3) all three mood dimensions energetic arousal, valence and calmness increased immediately after a training session (E: +10.4%; V: +17.8%; C: + 6.2%) but results were not significant and for arousal and calmness effect sizes were low. However, for valence we found a marginally significant effect with a high effect size. We assume that participation in a training session positively influenced acute valence of the *EG* participants. Morris and Salmon (1994) also provided evidence for positive changes in mood directly after running training. In line with the present study, Simons and Birkimer (1988) were unable to detect significant effects on tiredness. In contrast, Maroulakis and Zervas (1993) detected significant improvements in mood in all dimensions they investigated. Kanning et al. (2012) showed that daily life physical activity led to higher valence and energetic arousal in students immediately after the activity.

The heterogeneous mood effects pre to post-exercise of T1 (compared to T1–T3) possibly illustrate an interesting characteristic of inactive people. Acute exercise may lead to a decrease in mood at the beginning of an intervention and to an increase during the course of an intervention. For inactive people even moderate intensity endurance training leads to uncommonly high physiological efforts. An increased value of energetic arousal and a decreased value of calmness seem to support this assumption. Thus, inactive people may perceive exercise more aversive during the first sessions of an endurance intervention. The tendency of increased mood values of all three dimensions across the whole intervention period (T1–T3), indicates that in contrast to earlier findings (Steinberg et al., 1998), acute mood effects of acute exercise training differ from acute mood effects of regular exercise training. One can assume that if somebody is used to the physiological strain, regular exercise can rather positively influence acute calmness, energetic arousal and valence.

### Medium term effects of regular exercise

No medium term effects of regular aerobic endurance training on mood could be observed in the present study. The results are not in line with earlier studies in youths at risk (Lubans et al., 2012) and untrained men (Rimmele et al., 2007) showing that exercise and an active lifestyle, respectively led to benefits in psychological well-being.

The significant decrease of compliance at T3 illustrated that the criterion of at least six data entries per time of measurement was not achieved by several participants. Due to the low baseline endurance capacity of participants, endurance improvements could already be expected at T2 (after 6 weeks) (Hohmann et al., 2007). In addition, there were no important differences between T2 and T3. Thus, for the descriptive analyses of medium term effects of regular endurance training on mood, the data of T2 and T3 were combined. The results showing no medium term effects of regular exercise on mood during the first 8 weeks of training in the present study have to be interpreted with caution. Intra-individual progress over the course of the study could not be considered because average values were calculated based on available data entries. In addition, the sample size was not big enough because of the high number of missing data.

However, seven participants (3 *EG*, 4 *CG*) had at least eight data entries for each time of measurement. The descriptive analyses do not suggest meaningful changes in mood from T0 to T4.

Our study confirms results from Alfermann et al. (1993) and Moses et al. (1989). They could not find positive changes in habitual well-being as a result of regular physical activity and exercise.

In line with the results of the present study Steinberg et al. (1998) found decreased mood values over the course of the study while acute positive effects of exercise on mood were present. The findings underline that acute mood effects due to acute exercise have to be differentiated from a general mood enhancement through regular exercise. Alfermann et al. (1993) assumed that an intervention period of approximately half a year is not sufficient to influence global and enduring mental constructs. Berger and Motl (2000) point out that private and professional circumstances possibly have a greater influence than exercise intervention programs. Schlicht and Brand (2007) describe habitual mood as a very stable construct with limited ability to be changed. The young adults of the present sample faced the new situation of the apprenticeship may have had a greater influence on general mood in daily life compared to the positive influence of the regular endurance training.

According to Tellegen et al. (1988) genetic dispositions are one possible explanation. People tend toward a level of well-being that is typical for them, which is changed only briefly and within a more or less narrow corridor, even in the case of exposure to extreme events (Tellegen et al., 1988; Schlicht and Brand, 2007).

### Aerobic exercise capacity

The significant improvement of the diagnostic parameters of the progressive treadmill test from pre to post-exercise intervention showed that the endurance training achieved the pursued results. We achieved a good commitment of the participants to the regularly offered training sessions with an average participation of 14.25 ± 4.33 of a maximum 20 possible training sessions.

### Physical activity behavior

The objective activity monitoring during the whole study revealed several important aspects considering physical activity in both groups. The monitoring of the physical activity behavior of apprentices showed that they rarely pursue any moderate or intense exercise and that their daily routine primarily consists of low intensity activities or sedentary behavior.

During the intervention period, total activity intensity significantly differed between experimental and control group. The increase from 0 to 1% in the highest activity intensity level during the intervention period can be explained by the running training, as jogging episodes reveal a value of about 1000 mg/min (Kanning et al., 2012). Due to the decrease to 0.2% of time spent in this activity intensity level post the intervention, one can assume that no further increase in the highest activity intensity level besides jogging occurred. In addition, we could show that activity behavior of both groups did not change significantly pre to post-intervention period. This is displayed by the time spent in all other intensity levels which did not change in either *EG* or *CG*. During exercise programs one often faces the problem that the control group starts to become more active during the intervention period due to motivational reasons although they are not supposed to. Activity monitoring offers the potential to control for the activity of the control group.

Based on the results we assume that *EG* participants' daily life physical activity behavior did not impact the results of the exercise intervention. Mean activity intensity was higher post the intervention period in the *EG* (not significant) which may be an indicator that the intervention led to a higher daily physical activity level of the participants of the experimental group. To answer this question, future studies could add an assessment of physical activity few more weeks after the end of an exercise intervention.

### Ambulatory assessment of mood states and physical activity behavior

The compliance with accelerometers showed acceptable results. The participants did not feel disturbed by the sensor at any time. They sometimes forgot to put on the sensor in the morning and therefore the activity throughout the whole day could not be determined. For future studies a more interactive activity sensor emitting an acoustic signal or sending a message to a mobile phone if the sensor does not record activity, may limit this problem. This study showed that ambulatory activity monitoring helps to control physical activity behavior of control and experimental group during randomized controlled trials.

The assessment of subjective data via electronic diaries requires a certain self-discipline and willingness to answer the queries. Apparently it did not apply for the participants of the present study. Only a few participants accurately completed the data acquisition via the electronic diaries. Therefore, the question rises whether this approach of data acquisition is suitable for apprentices. Due to the decreasing data entries from T0 to T4, it seemed that the participants felt an increasing disinterest over the course of the study.

To adequately illustrate mood in daily life, more frequent mood assessments have to be conducted. Since mood is a very instable construct, single occasion questionnaires are not appropriate methods to address this issue. Ambulatory assessment offers a great potential to assess mood states across various situations and get more insights in the process of mood changes over time. Furthermore, recall bias can be reduced because data are collected when events actually happen. However, based on the poor compliance to mood assessments in this pilot study, more appropriate strategies have to be generated in future studies to address the question concerning repeated mood assessment via electronic diaries in this target group.

By installing a mobile phone application, participants would not have to carry an additional device (PDA) during the daily routine. Questions on current mood state could be asked relatively inconspicuously in this way.

Improvements of psychological wellbeing have been discussed in the context of health and adherence to physical activity. Positive mood values influence psychological health as an important part of general health of a person. People tend to choose and repeat behaviors they perceive as pleasant (Baumeister et al., 2007). The role of mood in decision making may also apply for exercise and thus positive effects of exercise on mood may enhance adherence to physical activity and exercise (Ekkekakis et al., 2011). The transition from school to an apprenticeship implicates life style changes in young people. Therefore, interventions to promote an active lifestyle may improve adherence to physical activity and serve as an important health preventive contribution.

Future studies should focus on the interaction between acute and medium term effects of exercise and mood in everyday life. Associations between daily life physical activity behavior and mood should be considered. Future studies should further investigate how acute mood effects differ between the beginning and the course of an endurance intervention in inactive people. To account for the characteristics of mood, more frequent measures are meaningful. Strategies to capture enough data to highlight intra-individual trends in effects of regular exercise on mood have yet to be determined.

### Conflict of interest statement

The authors declare that the research was conducted in the absence of any commercial or financial relationships that could be construed as a potential conflict of interest.

## References

[B1] Alfermann D. Lampert T. Stoll O. Wagner-Stoll P. (1993). Auswirkungen des sporttreibens auf selbstkonzept und wohlbefinden. Ergebnisse eines feldexperiments. Sportpsychologie 2, 21–27

[B2] Alfermann D. Stoll O. (1996). Befindlichkeitsveränderungen nach sportlicher Aktivität. Sportwissenschaft 26, 407–423

[B3] Baumeister R. F. Vohs K. D. DeWall C. N. Zhang L. (2007). How emotion shapes behavior: feedback, anticipation, and reflection, rather than direct causation. Pers. Soc. Psychol. Rev. 11, 167 10.1177/1088868307301033 18453461

[B4] Berger B. G. Motl R. W. (2000). Exercise and mood: a selective review and synthesis of research employing the profile of mood states. J. Appl. Sport Psychol. 12, 69–92 10.1080/10413200008404214

[B5] Cohen G. Java R. (1995). Memory for medical history. Accuracy of recall. Appl. Cogn. Psychol. 9, 273–288 10.1002/acp.2350090402

[B6] Dickhuth H. H. Mayer F. Röcker K. Berg A. (2007). Sportmedizin für Ärzte. Köln: Deutscher Ärzteverlag

[B7] Ekkekakis P. Parfitt G. Petruzzello S. J. (2011). The pleasure and displeasure people feel when they exercise at different intensities. Sports Med. 41, 641–671 10.2165/11590680-000000000-00000 21780850

[B8a] Ekkekakis P. Hall E. E. Van Landuyt L. M. Petruzzello S. J. (2000). Walking in (affective) circles: can short walks enhance affect? J. Behav. Med. 23, 245–275 10863677 10.1023/a:1005558025163

[B8] Fahrenberg J. Leonhart R. Foerster F. (2002). Alltagsnahe Psychologie. Datenerhebung im Feld mit hand-held PC und physiologischem Mess-System. Bern: Verlag Hans Huber

[B9] Fahrenberg J. Myrtek M. Pawlik K. Perrez M. (2007). Ambulatory assessment: monitoring behavior in daily life settings. Eur. J. Psychol. Assess. 23, 206–213 10.1027/1015-5759.23.4.206

[B10] Härtel S. Gnam J.-P. Löffler S. Bös K. (2011). Estimation of energy expenditure using accelerometers and activity-based energy-models-validation of a new device. Eur. Rev. Aging Phys. Act. 8, 109–114 10.1007/s11556-010-0074-5

[B11] Heck H. Mader A. Hess G. Mücke S. Müller R. Hollmann W. (1985). Justification of the 4 mmol/L lactate threshold. Int. J. Sports Med. 6, 117–130 10.1055/s-2008-1025824 4030186

[B12] Hohmann A. Lames M. Letzelter M. (2007). Einführung in die Trainingswissenschaft (4., überarb. und erw. Aufl.). Wiebelsheim: Limpert

[B13] Hufford M. R. Shiffman S. Paty J. Stone A. A. (2001). Electronic momentary assessment: real- world, real-time measurement of patient experience, in Progress in Ambulatory Assessment. Computer-Assisted Psychological and Psychophysiological Methods in Monitoring and Field Studies, Chapter 4, eds Fahrenberg J. Myrtek M. (Göttingen: Hogrefe and Huber), 69–92

[B14] Kanning M. Ebner-Priemer U. W. Brand R. (2012). Autonomous regulation mode moderates the effect of actual physical activity on affective states: an ambulant assessment approach to the role of self-determination. J. Sport Exerc. Psychol. 34, 260–269 22605366 10.1123/jsep.34.2.260

[B15] Kanning M. Schlicht W. (2010). Be active and become happy: an ecological momentary assessment of physical activity and mood. J. Sport Exerc. Psychol. 32, 253–261 20479481 10.1123/jsep.32.2.253

[B16] Kubukeli Z. N. Noakes T. D. Dennis S. C. (2002). Training techniques to improve endurance exercise performances. Sports Med. 32, 489–509 10.2165/00007256-200232080-00002 12076176

[B17] Lubans D. R. Plotnikoff R. C. Lubans N. J. (2012). Review: a systematic review of the impact of physical activity programs on social and emotional well-being in at-risk youth. Child Adolesc. Ment. Health 17, 2–13 10.1111/j.1475-3588.2011.00623.x 32847310

[B18] Maroulakis E. Zervas Y. (1993). Effects of aerobic exercise on mood of adult women. Percept. Mot. Skills 72, 795–801 10.2466/pms.1993.76.3.795 8321589

[B19] Morris M. Salmon P. (1994). Qualitative and quantitative effects of running on mood. J. Sports Med. Phys. Fitness 34, 284–291 7830393

[B20] Moses J. Steptcoe A. Mathews A. Edwards S. (1989). The effects of exercise training on mental well-being in the normal population: a controlled trial. J. Psychosom. Res. 33, 47–61 10.1016/0022-3999(89)90105-0 2926699

[B21] Reed J. Buck S. (2009). The effect of regular aerobic exercise on positive activated affect: a meta-analysis. Psychol. Sport Exerc. 10, 581–594 10.1016/j.psychsport.2009.05.009

[B22] Reed J. Ones D. S. (2006). The effect of acute aerobic exercise on positive activated affect: a meta-analysis. Psychol. Sport Exerc. 7, 477–514 10.1016/j.psychsport.2005.11.003

[B23] Rimmele U. Zellweger C. B. Marti B. Seiler R. Mohiyeddini C. Ehlert U. (2007). Trained men show lower cortisol, heart rate and psychological responses to psychosocial stress compared with untrained men. Psychoneuroendocrinology 32, 627–635 10.1016/j.psyneuen.2007.04.005 17560731

[B24] Ross C. Hayes D. (1988). Exercise and psychological well-being in the community. Am. J. Epidemiol. 127, 762–771 3258471 10.1093/oxfordjournals.aje.a114857

[B25] Schlicht W. Brand R. (2007). Körperliche Aktivität, Sport und Gesundheit. Eine interdisziplinäre Einführung. Weinheim: Juventa

[B26a] Schwerdtfeger A. Eberhardt R. Chmitorz A. (2008). Gibt es einen Zusammenhang zwischen Bewegungsaktivität und psychischem Befinden im Alltag? Z. Gesundheitspsychol. 16, 2–11 10.1026/0943-8149.16.1.2

[B26] Shiffman S. Stone A. Hufford M. R. (2007). Ecological momentary assessment. Annu. Rev. Clin. Psycho. 4, 1–32 10.1146/annurev.clinpsy.3.022806.091415 18509902

[B27] Simons C. W. Birkimer J. C. (1988). An exploration of factors predicting the effects of aerobic conditioning on mood state. J. Psychosom. Res. 32, 63–75 10.1016/0022-3999(88)90089-X 3404491

[B28] Steinberg H. Nicholls B. R. Sykes E. A. LeBoutillier N. Ramalakhan N. Moss T. P. (1998). Weekly exercise consistently reinstates positive mood. Eur. Psychol. 3, 271–280 10.1027/1016-9040.3.4.271

[B29] Stephens T. (1988). Physcal activity and mental health in the United States and Canada: evidence from four population surveys. Prev. Med. 17, 35–47 10.1016/0091-7435(88)90070-9 3258986

[B30] Stone A. A. Shiffman S. Schwartz J. E. Broderick J. E. Hufford M. R. (2003). Patient compliance with paper and electronic diaries. Control. Clin. Trials 24, 182–199 10.1016/S0197-2456(02)00320-3 12689739

[B31] Tanisho K. Hirakawa K. (2009). Training effects on endurance capacity in maximal intermittent exercise: comparison between continuous and interval training. J. Strength Cond. Res. 23, 2405–2410 10.1519/JSC.0b013e3181bac790 19826281

[B32] Tellegen A. Lykken D. T. Bouchard T. J. Wilcox K. J. Segal N. L. Rich S. (1988). Personality similarity in twins reared apart and together. J. Pers. Soc. Psychol. 54, 1031–1039 10.1037/0022-3514.54.6.1031 3397862

[B33] Wilhelm P. Schoebi D. (2007). Assessing mood in daily life. structural validity, sensitivity to change, and realiability of a short-scale to measure three basic dimensions of mood. Eur. J. Psychol. Assess. 23, 258–267 10.1027/1015-5759.23.4.258

[B34] Zintl F. (1997). Ausdauertraining - Grundlagen, Methoden, Trainingssteuerung, 4. Aufl. München: Verlag BLV

